# Using Magnetic Resonance Imaging During Childbirth to Demonstrate Fetal Head Moldability and Brain Compression: Prospective Cohort Study

**DOI:** 10.2196/27421

**Published:** 2022-11-30

**Authors:** Olivier Ami, Jean-Christophe Maran, Dominique Musset, Claude Dubray, Gérard Mage, Louis Boyer

**Affiliations:** 1 Clinique de la Muette - Ramsay Santé Paris France; 2 Image Guided Therapies Institut Pascal, UMR 6602 Université Clermont Auvergne and CNRS Paris France; 3 Université Paris Sud 11 Paris France; 4 Centre d'investigations cliniques Université Clermont Auvergne Clermont Ferrand France; 5 Service de gynécologie-obstétrique Université Clermont Auvergne Clermont Ferrand France; 6 Pole radiologie CHU Clermont Ferrand Gabriel Montpied Clermont-Ferrand France

**Keywords:** parturition, magnetic resonance imaging, obstetrics, fetus, cephalopelvic disproportion

## Abstract

**Background:**

Childbirth is a physiological process with significant medical risk, given that neurological impairment due to the birthing process can occur at any time. Improvements in risk assessment and anticipatory interventions are constantly needed; however, the birthing process is difficult to assess using simple imaging technology because the maternal bony pelvis and fetal skeleton interfere with visualizing the soft tissues. Magnetic resonance imaging (MRI) is a noninvasive technique with no ionizing radiation that can monitor the biomechanics of the birthing process. However, the effective use of this modality requires teamwork and the implementation of the appropriate safeguards to achieve appropriate safety levels.

**Objective:**

This study describes a clinically effective and safe method to perform real-time MRI during the birthing process. We reported the experience of our team as part of the IMAGINAITRE study protocol (France), which aimed to better understand the biomechanics of childbirth.

**Methods:**

A total of 27 pregnant women were examined with 3D MRI sequences before going into labor using a 1-Tesla open-field MRI. Of these 27 patients, 7 (26%) subsequently had another set of 3D MRI sequences during the second stage of labor. Volumes of 2D images were transformed into finite element 3D reconstructions. Polygonal meshes for each part of the fetal body were used to study fetal head moldability and brain compression.

**Results:**

All 7 observed babies showed a sugarloaf skull deformity and brain compression at the middle strait. The fetus showing the greatest degree of molding and brain shape deformation weighed 4525 g and was born spontaneously but also presented with a low Apgar score. In this case, observable brain shape deformation demonstrated that brain compression had occurred, and it was not necessarily well tolerated by the fetus. Depending on fetal head moldability, these observations suggest that cephalopelvic disproportion can result in either obstructed labor or major fetal head molding with brain compression.

**Conclusions:**

This study suggests the presence of skull moldability as a confounding factor explaining why MRI, even with the best precision to measure radiological landmarks, fails to accurately predict the modality of childbirth. This introduces the fetal head compliance criterion as a way to better understand cephalopelvic disproportion mechanisms in obstetrics. MRI might be the best imaging technology by which to explore all combined aspects of cephalopelvic disproportion and achieve a better understanding of the underlying mechanisms of fetal head molding and moldability.

## Introduction

### Background

Events occurring within the first few hours of birth can cause fetal cerebral palsy or maternal perineum trauma, determining a human’s life trajectory. There is an urgent need for predictive and preventive tools to prevent avoidable birth traumas. Magnetic resonance imaging (MRI) is a noninvasive and nonirradiating tool suitable for exploring the biomechanics of the birthing process and evaluating fetal well-being. A clinical protocol to achieve this goal requires numerous safeguards to ensure the same level of safety in the MRI suite as in the delivery room.

In 1948, Mengert [1] described five components of cephalopelvic disproportion: (1) size and shape of the bony pelvis, (2) size of the fetal head, (3) force exerted by the uterine contractions, (4) moldability of the fetal head, and (5) presentation and position of the fetal head. A sixth component with intrapelvic organs and structures shaping the birth canal might also be considered [2]. Exploring these factors with imaging during birth is crucial to the prediction and prevention of cephalopelvic disproportion.

MRI is routinely used to explore fetal malformations antenatally and perform obstetric pelvimetry [3]. This technique, which is neither invasive nor irradiating, is safe for the birthing person and the fetus [4-9]. Parturients near term usually require an open-field MRI or a large oval bore, given their increased abdominal circumference. However, despite the potential advantages of MRI when evaluating the birthing process in real time, we identified only a few sites worldwide with an open-field MRI near a maternity ward that would enable the exploration of childbirth in all positions.

### Prior Work

In 2010, Dr Christian Bamberg (Charité Hospital, Berlin) demonstrated the feasibility of MRI in childbirth [10,11] in response to a clear need to observe images of this process, which had only previously been described by obstetricians. Since then, MRI has made an enormous progress, given that it offers high-resolution 3D imaging and can evaluate the properties and composition of various tissues. Magnetic resonance (MR) elastography techniques are now available to measure tissue elasticity [12], and diffusion MRI explores water molecule movement in cells and tracks fibers on fetal cerebral nerve cells, uterine cells, and perineal muscle cells [13,14].

MR spectroscopy can explore tissue composition while acquiring qualitative and quantitative information on the presence of various molecules such as lactates [15-17], their tissue distribution, and changes in their concentrations over time. Multinuclear MR spectroscopy can explore fetal energy reserves in models of chronic fetal distress [18]; it can also study the maturity of fetal organs [19] as part of prematurity studies or in the exploration of certain metabolic diseases or birthing person-to-fetus drug transmission. Functional brain MRI with blood oxygen level–dependent imaging [20-22] may also be used, whereas proton spectroscopy can be used to assess lactate levels to examine the health of fetal tissues and the placenta [23].

In the short term, information available from 3D MRI during the birthing process can simulate childbirth and facilitate virtual reality birthing tests that are closer to reality than pelvimetry [24,25]. Moreover, a better understanding of fetal head compression could explain spontaneously observed cerebral hemorrhages and fetal heart rate abnormalities that can occur during the birthing process [26-28]. Thus, MRI can reveal important information that could be used at this crucial time.

### Goal

We report our team’s experience with the IMAGINAITRE research protocol (France), which was approved by French authorities and a French National Ethics Committee. We aimed to observe the biomechanical changes of the fetal head and maternal perineum during human birth [2,29]. This paper describes a clinically effective and safe method for both the birthing person and child to perform real-time MRI during the birthing process.

## Methods

### Research Protocol

This research protocol was developed by the University Hospital of Clermont-Ferrand (Auvergne University) to meet specific research objectives and identify suitable locations to conduct this research in France. In our study, a public or private partnership emerged because of the location of and the equipment available at the private establishment that hosted this protocol. This private institution, located in Evry (Hopital Privé de l’Essonne, F91000, France), had clinical permits to operate both a maternity ward and a radiology department with open-field MRI in the same building.

### Ethical Considerations

This prospective biomedical interventional study (IMAGINAITRE) was approved by the French ethical institutional review board “Ile de France II” (ID-RCB 2012-A01469-34) and the French National Agency for Drug and Medical Product Safety (*Agence Nationale de Sécurité du Médicament et des Produits de Santé*) and promoted by the University of Clermont-Ferrand Medical Center. All the women agreed to participate and signed an informed consent.

### Preliminary Organization of Clinical and Radiological Teams

Performing an MRI during childbirth requires close collaboration among the clinical teams that usually support the patient in the delivery room. The birthing process is unpredictable in onset and duration; pregnant patients can deliver at any time, which does not correspond well with the scheduled studies of most radiology departments. It is important that both the obstetric and radiology teams are comfortable with performing the required MRI protocol safely and that patient management remains focused on the birth and not on obtaining images. It is necessary that the team dedicated to obtaining images be available 24 hours a day, 7 days a week, which requires close cooperation between the imaging and maternity departments; an on-call list of staff should be dedicated to this activity. All members of the research protocol team should be trained and ready to make themselves MR safe (ie, by removing any metal or devices from their body and selecting only nonmagnetic, MR-safe equipment), should they be required to enter the magnetic field to perform any emergency procedure.

The delivery suite should be located near the imaging suite to allow staff to carefully monitor the patient during transfer and throughout the examination. A formal protocol should exist within each department outlining the equipment needed for patient transfer between the departments, including a dedicated stretcher, a portable epidural kit with a syringe pump and remote bolus, a sterile protection equipment and aseptic products, obstetric instruments, and a nonmagnetic delivery kit.

The staff dedicated to the protocol must remain with the patient from the time they leave the delivery room. The obstetrician and anesthesiologist must always remain at the patient’s side. The other parent’s presence during transfer is often reassuring for the parturient in labor. The transfer time between the departments must not exceed a few minutes; fetal monitoring should be performed continuously. If battery monitoring tools are used, monaural stethoscopes must be available should the electronic devices fail. The transfer of the patient to the MRI suite should not occur if there are signs of fetal distress, an abnormal heart rhythm, clear amniotic fluid, or any pain or other abnormalities noted during labor. An obstetrician or midwife should perform an examination before departure, according to the strict protocol.

A dedicated operating room must be available before the patient’s departure from the obstetric suite and throughout the imaging procedure; staff should be trained and provided with the appropriate equipment to perform the protocol and the requisite emergency procedures. These research activities must have ethics and institutional approval, and they must also be covered by insurance.

### Patient Selection

The inclusion criteria are as follows: parturients who are in the third trimester; who are aged 21 to 39 years; who are primipara, secundipara, or tertipara; who are with no known risk factors that can affect delivery; and who have provided their informed consent to participate. The exclusion criteria include fetal breech presentation or presentation that is not strictly cephalic at the time of delivery, a scarred uterus, multiple pregnancy, known maternal or fetal medical conditions or those requiring urgent care, and a fetal heart rate requiring care every 30 minutes. The contraindications for open-field MRI are as follows: a ferromagnetic foreign body in the patient’s body, claustrophobia, parturients aged <21 years, a vulnerable adult, an abnormal fetal heart rate requiring treatment within 30 minutes, contraindications to MRI, or no prior obstetrical disease during pregnancy.

The patients are selected in advance during pregnancy monitoring and are asked about contraindications to MRI (ie, claustrophobia and ferromagnetic foreign bodies). The physician then discusses the research protocol with the couple and arranges a visit to the MRI, maternity ward, and imaging services. The patients review, sign, and return the consent forms if they agree to participate.

The patient is then invited to a prepartum MRI examination on the date closest to the start of the term period to establish a baseline examination; ideally, the other parent is present so that the patient can experience the imaging protocol as it will take place during labor, which usually lasts only 3 to 5 minutes. Any remaining questions are addressed, and the signed consent forms are obtained. Participants’ charts are indicated with a label unique to them.

### Clinical Support When Leaving the Delivery Room

An MRI-specific delivery procedure is displayed in the delivery room. When the enrolled patient goes into spontaneous labor and enters the delivery room, the on-duty midwife calls the protocol coordinator. The progress of the labor can be monitored by the local on-call team until dilation is achieved. If imaging is scheduled to take place during the second phase of labor, the entire team should arrive before the patient reaches cervical dilation no greater than 8 cm. Once the entire team is on site, the obstetrician examines the patient to confirm presentation height and cervical dilation.

**Figure 1:**
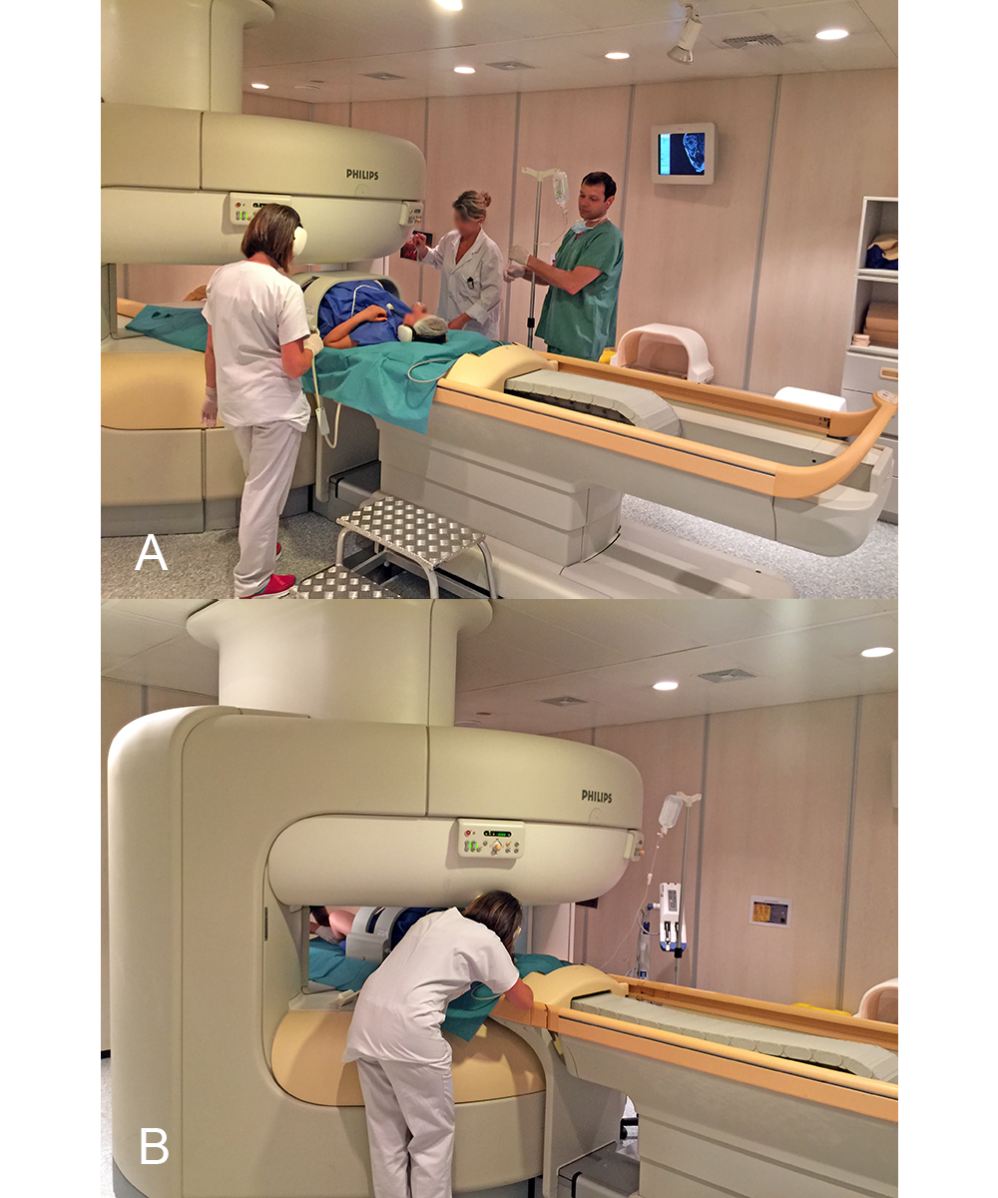
(A) Open magnetic resonance imaging (MRI) room with sterile drapes and a delivering patient. (B) Patient in the open-field MRI after antenna installation and being positioned inside the magnetic field.

Once the second stage of labor is reached, an anesthetic epidural bolus is administered, and the patients are asked not to push; they are then transferred to the MRI table. In our hospital, the average transfer time between the bed in the delivery room and the MRI table is 2 minutes and 36 seconds. Once the patient is in the MRI suite, the nonmagnetic transport stretcher is positioned parallel to the MRI table outside the range of the 5-Gauss line. Sealed and sterile protections are placed on the MRI table (Figure 1).

### Imaging Process

**Figure 2:**
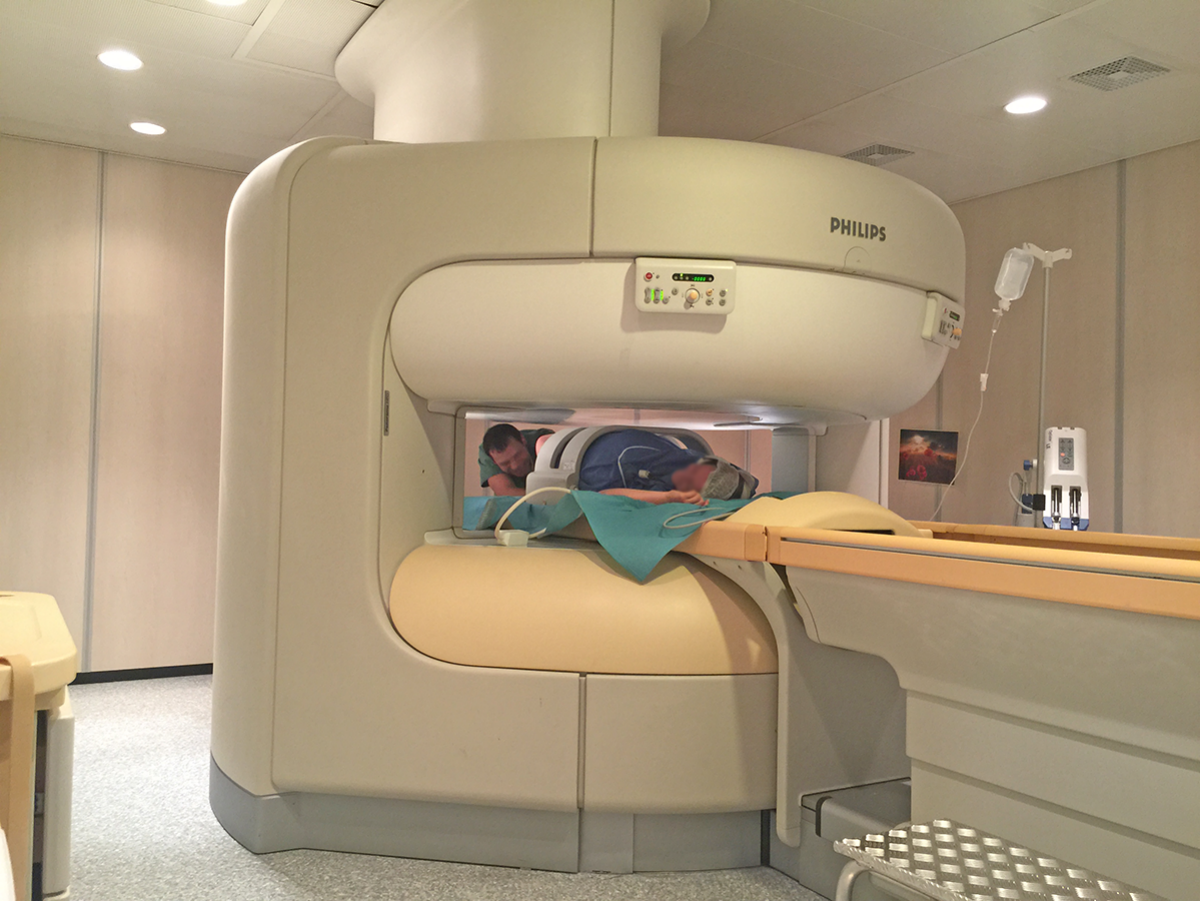
Patient installed in the open-field magnetic resonance imaging, with a gynecologist by her side.

The patient is settled on the nonmagnetic stretcher for transfer to the MRI machine. After the administration of a last epidural bolus, the epidural catheter can be closed by a plastic cap. The patient is then positioned in the MRI machine with a surface or bridge antenna in the open-field MRI, enabling mobility. The bearing areas should be protected by padded reinforcements; the patient should be comfortable (Figure 2). The patient wears earplugs and ear protectors to reduce the scanner acoustic noise. The antenna is centered on the pelvic region, which is wide enough to get the signal below the perineum and back to the top of the uterus. The patient is provided with preliminary instructions; a first localization examination is performed. The sequences are then implemented with centering in the space.

**Figure 3:**
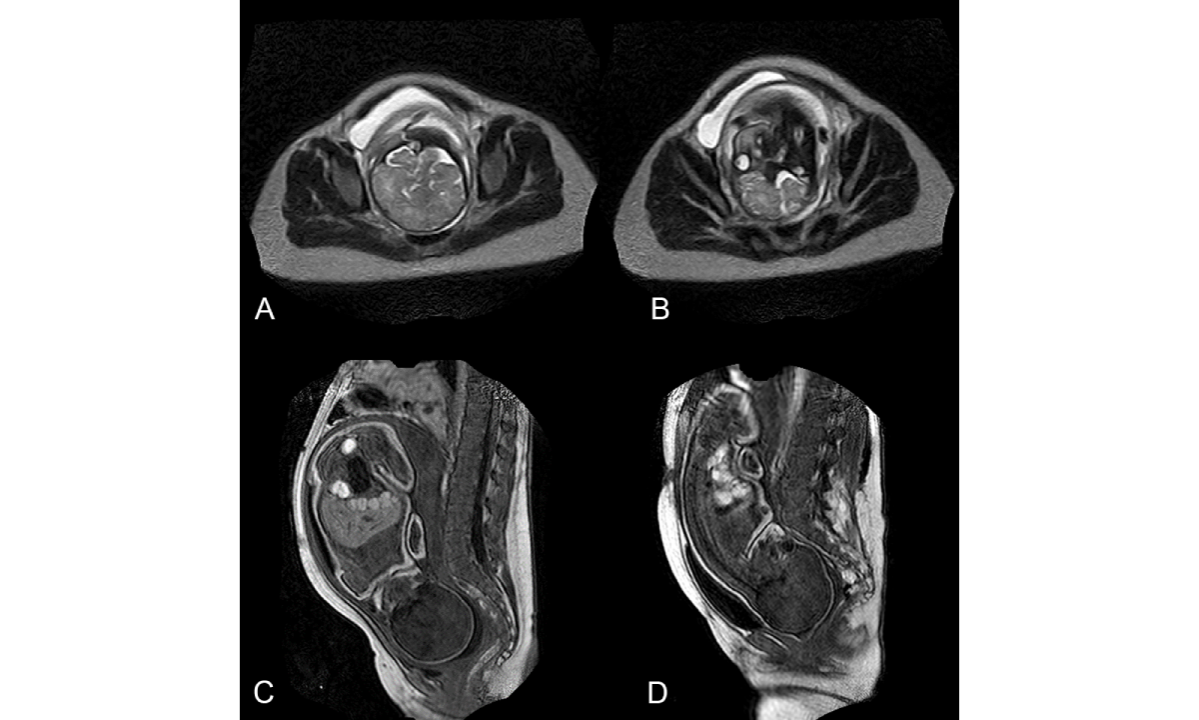
(A) T2 gradient echo sequence showing an axial slice centered on the fetal brain at the superior inlet level. (B) The same sequence at the iliac bone level. (C) T1 gradient echo sequence in the sagittal plane showing bony pelvis and fetal head before entering into labor. The empty bladder is in retropubic position. (D) Same T1 gradient echo sequence showing the fetal head molding in the middle brim during second phase of labor with a full bladder. The fetal head is rotated in occipito-pubic position, and the full bladder is ascended above the upper limit of the pubic bone.

**Figure 4:**
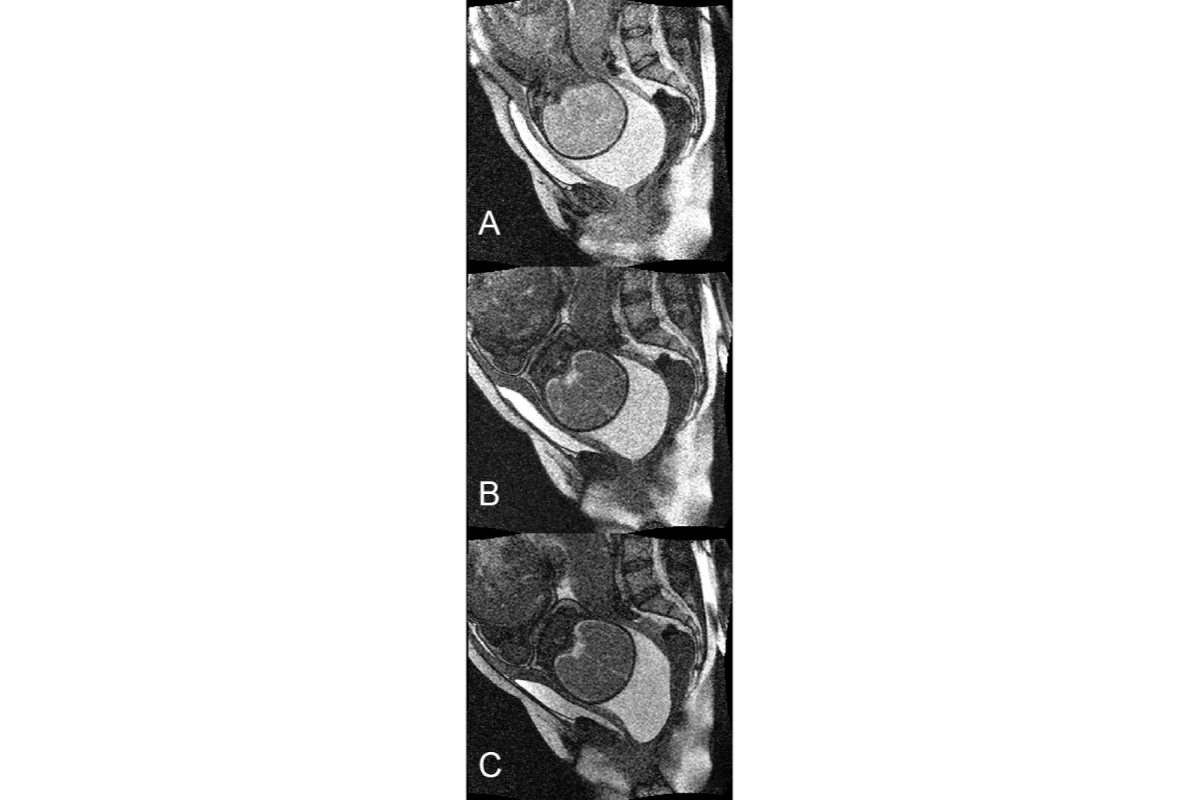
T2 dynamic imaging during maternal effort (successive images) at complete cervix dilation and unruptured membranes (dynamic acquisition). (A) Before maternal efforts: fetal head is above the sacro-pubic line. (B) Beginning of maternal efforts: the membranes start to change form to underline the vaginal route. (C) The intact membranes are approaching the superficial perineum level inside the vagina, the coccygeal bone is pushed posteriorly, the fetal head is under the superior limit of the pubic bone, and the bladder and the urinary sphincter are descending with the fetal head.

The imaging protocol may be adjusted as needed. The MRI sequences should be optimized beforehand with an application engineer to obtain fine contiguous images that balance resolution and acquisition speed. The region of interest should be broad, and the number of excitations is limited to the acquisition of a correct signal. The use of the rapid-filling technique in the Fourier plane and a superconducting antenna significantly reduces acquisition times. The fetus moves very little in the pelvis; however, diaphragmatic movements are more significant because of maternal breathing. Therefore, it is advisable to ask the birthing person to maintain apnea when acquiring images of the subcostal region. Once static acquisitions are obtained in the 3 planes in the decubitus dorsal position in T1 gradient echo throughout the uterine contents and just below the perineum, rapid-sequence, contiguous T2 images centered on the fetal brain and the birthing person’s perineum are performed (Figure 3). A dynamic sequence can then be launched in true fast imaging with steady state precession in which 1 image per second is obtained for 60 seconds along the sagittal and frontal planes at the base of the iliococcygeal head of the levator ani; this informs the movement of various muscle groups or other organs during pushing (Figure 4). 3D reconstructions can be adjusted, as the contours observed in 2D can be altered when a change is observed in a region of interest. Posture changes can then be made with a new 3D, T1 gradient echo in the 3 planes; these scans can observe the modification of the trajectory of the fetal head as a consequence of postural change. Furthermore, the delivery process can be observed by dynamic imaging or the acquisition of successive static volumes.

### Foreseeable Risks and Emergency Protocols

#### Overview

According to the legal provisions in France, maternal and fetal exposure to magnetic fields must not exceed 2 Tesla for temporary, whole-body exposure. No adverse effects have been reported since MRI exists and has been used worldwide during pregnancy [30]. Given the potential adverse effects of gadolinium on fetuses, the use of MR contrast media is not recommended for parturients [31].

We used an open-field MRI machine low-magnetic field (1T; Phillips Panorama). The measurement of the cumulative maternal specific absorption rate should remain below the maximum exposure recommendations. For comfort and tolerance, we do not recommend using MRI in parturients for more than 90 minutes [32-34]. In our protocols, we never exceeded more than 15 minutes of cumulative examination time.

There are 2 types of foreseeable risks during such protocols: those related to the use of MRI and those unrelated to the use of MRI. Risks associated with the use of an MRI machine in parturients during labor are detailed below.

#### Risks Related to the Use of MRI

##### Claustrophobia

Anxiety and agitation related to the phobia of enclosed spaces is possible, although this type of anxiety is usually mild and quickly resolved. These incidents are typically mitigated by using an open-field device and by short imaging sequences. The patient can be quickly and easily removed from the MRI room with staff standing nearby. To anticipate and mitigate this risk, pelvic MRI should be scheduled during the eighth or ninth month of pregnancy; this allows patients to become familiar with the MRI procedure and meet the protocol team. Any questions or concerns can be addressed to enhance the patients’ understanding of the study.

##### Scanner Acoustic Noise to the Birthing Person and Fetus

The intensity of the sound wave generated by the MRI machine depends on the amplitude of the vibrations transmitted to the coils by the geometry of the sequence, which means that the amount of noise depends on the sequence type. The noisiest sequence with the Philips Panorama 1T MRI machine is the *real-time balanced TFE* at 108 dB, whereas the gradient echo T1 and T2 sequences proposed to image the birthing process generate approximately 90 to 95 dB. Temporary exposure of the public in France is limited to an average intensity of 105 to 120 dB peak sound pressure to prevent hearing damage.

For the birthing person, the sound level related to MRI sequence acquisition is reduced by using earplugs or ear protection, which are put in place before entering the machine. For the fetus, scanner acoustic noise can be perceived with intact or ruptured membranes.

###### Intact Membranes

The fetus is surrounded by amniotic fluid; sound waves reaching here initially propagate in the air. If a planar sound wave propagates without weakening in medium 1 and passes through medium 2, the sound intensity generally decreases. According to Snell law, with the passage of sound waves from air to water, the fetus is protected from scanner acoustic noise in its amniotic fluid, given that the sound intensity transmitted to the fetus is on the order of 0.12% of the incident intensity.

###### Ruptured Membranes

Ruptured membranes occur in cases where the cervix starts to expand and the patient is in labor. Here, the lower segment is extended, and the myometrium in the lower segment is molded on the fetal head. Owing to the proximity of the impedance of the biological tissue to water, we are only interested in reducing scanner acoustic noise by blocking the fetus’s ears in the lower segment, where sound is directly transmitted to the eardrum through fetal aerial vibration. In this case, the geometry of the acoustic wave is highly attenuated by the fact that the fetus is in a sealed enclosure: a little amniotic fluid is often trapped over the fetus’s head, as the lower segment molds the cephalic pole.

Acoustic attenuation depends on the thickness of the maternal tissues that protect the fetal eardrum from scanner acoustic noise. Although this has never been measured in utero, the calculation of noise reduction was at least 30 dB, which would reduce the noise from 95 to 65 dB for about 10 minutes, which is less than the noise made by a domestic vacuum cleaner. There are no adverse consequences to the unborn child’s hearing; however, when the child is born, the absence of tympanic protection requires the sequences can be continued after the use of specific hearing protection when the ears are viewed at the level of the vulva.

#### Risks Not Related to the Use of MRI

In the event of unexpected maternal discomfort, the imaging procedure is stopped immediately, and the anesthesiologist can provide immediate care. All equipment required for resuscitation is available throughout the procedure, as is an operating room, if required.

If membrane rupture occurs during the examination, it can cause the sudden emission of a stream of amniotic fluid. Therefore, the exposed MRI surfaces are protected by disposable, sterile, and waterproof drapes. Furthermore, if delivery occurs during the MRI scan, a dedicated midwife and obstetrician (in addition to the clinical care team) are present in the MRI room. All equipment necessary to manage childbirth in the MRI room is readily available (plastic disposable material, nonmagnetic forceps and scissors, drapes, and compresses), including a nonferromagnetic plastic suction cup if an instrumental delivery is required.

Finally, there could be instances in which maternal or fetal emergency or both occur during the examination. Three situations can occur: cord prolapse, abnormal fetal heart rate with the risk of acidosis, and sudden bleeding. To detect these risks, the entire obstetric team will remain with the patient at every stage of the imaging procedure. An operating room to perform a cesarean section and emergency extraction is available for the duration of the procedure. This operating room is located such that the child’s extraction can be guaranteed within 10 minutes of problem detection. Furthermore, fetal heart rate and uterine contractions are monitored during patient transfer and throughout the procedure using a portable, low-current measurement device. If a suitable fetal heart rate monitoring device is not available in the MRI suite, intermittent auscultation can be used for a cumulative period not exceeding 10 minutes.

Patients participating in the protocol will be transferred to the MRI room only if an effective epidural is administered and dosed correctly. An anesthesiologist dedicated to the protocol will be on site to administer the epidural and maintain optimal levels of pain relief.

### Image Postprocessing Method

**Figure 5:**
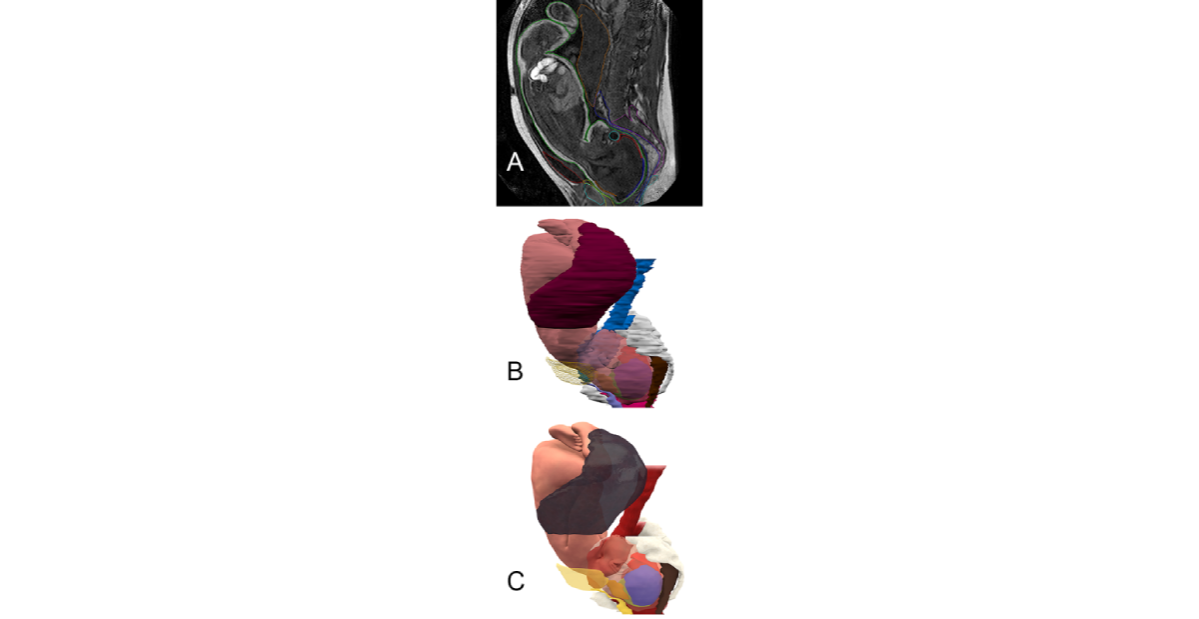
3D reconstruction and finite element meshing. (A) 2D image with mesh contouring. (B) Artifacted 3D mesh. (C) 3D volumes after optimization process.

**Figure 6:**
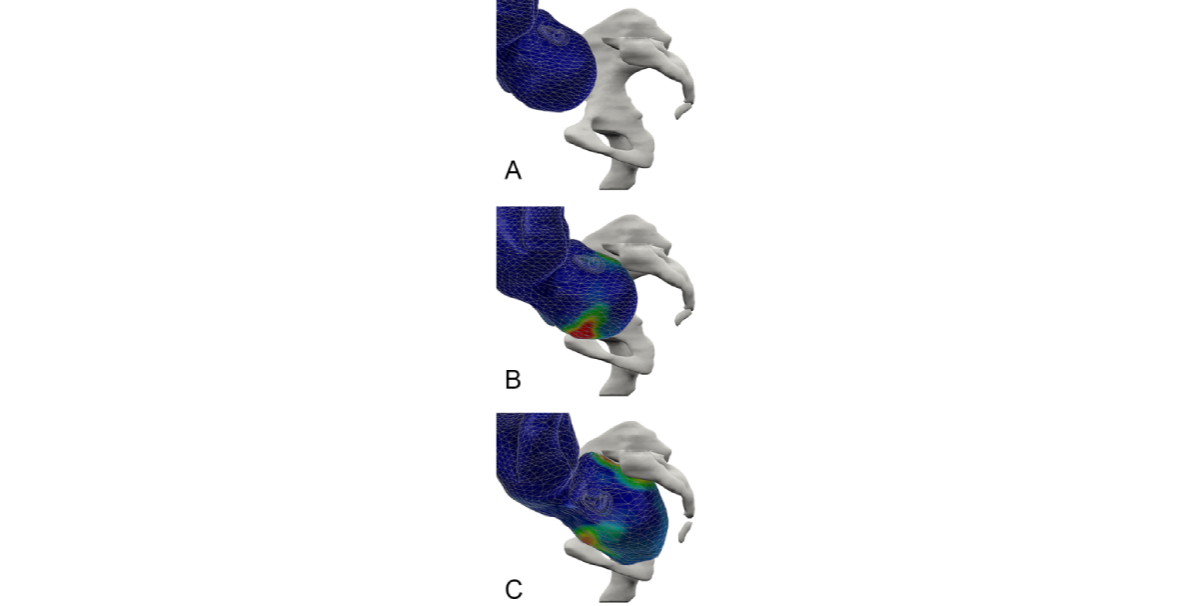
3D fetal mesh in false colors (blue) showing the forces applied on the fetal head and their gradient (the greatest forces are displayed in hot colors) from left to right. (A) Head is above the superior inlet. (B) The fetal head is engaging at the superior brim with a contact of the occiput on the pubic bone. (C) The fetal head is engaging at the inferior brim and shows an important fetal head molding.

We recommend using a 3D finite element postprocessing station. This can aid in the manual or automated reconstruction of the vector contours of the fetal organ and birth canal surfaces (Figure 5). Finite elements meshing thus permits the removal of most imaging-related artifacts. Vector meshing can be printed in 3D or exported for use in downstream imaging simulation software. A 3D fetal mesh can then be used for biomechanical studies and to retro-evaluate forces applied to the fetal head (Figure 6).

## Results

### Outcomes Description

Of the 27 patients who agreed to participate in the protocol, 7 (26%) presented with conditions that allowed for an MRI during the second phase of labor. The 7 patients were, on average, aged 28 (range 23-34) years ; 3 (43%) were primipara, 3 (43%) were secundipara, and 1 (14%) was tertipara. Moreover, of these 7 patients, 3 (43%) were beyond 39 weeks of gestation, and 4 (57%) were past their due date. The membranes were ruptured in 86% (6/7) of the patients, and the patients were at rest during the MRI scans, just before pushing.

The Magnin index was calculated by adding the obstetric conjugate diameter to the median transverse diameter.

The median Magnin score of the 7 patients with imaging during the second stage of labor was 26 (IQR 25.1-27.5). None of the patients in our series showed any modification of pelvic diameters at the superior brim during the second phase of labor, in comparison with the MRI acquired before entering into labor.

The newborns had a median weight of 3755 (IQR 3095-4525) g, and all 10-minute Apgar scores were 10.

In total, 71% (5/7) of babies were born by natural delivery without instrumental extraction, and 29% (2/7) were born by emergency cesarean section because of stagnation when fully dilated, of which 1 (14%) was after forceps failure. In both cases, the pregnancies were overdue.

No patient had pelvic dystocia predictable from the size of their pelvis, given that all Magnin scores were above 25.

### Rotation During Labor

Of the 7 observed babies, 6 (86%) rotated to face their placentas during labor, driven by contractions, with the convexity of the baby’s back responding to the smooth concavity of the uterus.

### Bladder Behavior

The bladder of all birthing persons who retained a postmicturition residue before delivery reascended above the pubic bone plane at the time of expulsive efforts. When emptied, the bladder fell below the pubic bone plane and appeared at risk of being dragged by rubbing between the immobile bony wall of the pelvis and the fetal head (Figure 3).

### Fetal Skull Compliance and Brain Compression

All 7 observed babies showed a sugarloaf skull deformity and a brain compression at the middle strait. This deformation remained visible in only 29% (2/7) of babies immediately after delivery. During this deformation, a sequence involving the skull bones, fontanel closure, tentorium cerebelli, and falx cerebri was initiated, resulting in movements of cerebrospinal fluid from the lateral ventricles and brain periphery toward the skull base and large cistern. All 7 observed babies showed cerebrospinal fluid distributed around the cerebellum during the fetal skull molding at the middle brim.

The fetus showing the greatest degree of molding and brain shape deformation weighed 4525 g and was born spontaneously but also presented with a low Apgar score.

## Discussion

### Principal Findings

In the case of the fetus with the greatest degree of fetal skull molding, observable brain shape deformation demonstrated that brain compression occurred, and it was not necessarily well tolerated by the fetus, which illustrates the moldability criteria described by Mengert [1]. This observation, far from ruling out a cephalopelvic disproportion, suggests the presence of skull moldability as a confounding factor explaining why MRI, even with the best precision to measure radiological landmarks, fails to accurately predict the modality of childbirth. This introduces the fetal head compliance criterion as a way to better understand cephalopelvic disproportion mechanisms. This observation is probably the most remarkable, given that it allows the rediscovery of the wealth of scientific work performed on the phenomenon of static brain compression of the child during the labor and delivery processes [35-39], and revives the notion of fetal head compliance in the interpretation of cephalopelvic disproportion situations at birth.

The fact that the cerebrospinal fluid moves to the posterior fossa during the shaping of the fetal skull is another interesting finding on the shock-absorber role that this fluid can play, and the circulation channels that are responsible for these hydraulic movements. During this process, the subarachnoid spaces widen in the posterior fossa.

The rotation of infants facing their placenta during labor was also an interesting finding in this work. It appears that the placenta is a major determinant of the presentation of the child to the upper strait engagement, whose position can be determined well in advance. Whether the risk of occipito-sacral presentation can be effectively predicted when the placenta is anterior, for example, should be investigated in the future. This would allow for more complete maternal information about the upcoming delivery to be given early in the second trimester.

Finally, the important logistics realized by our MRI during patients’ labor allowed us to observe the behavior of the bladder, which had filled by the time the imaging was performed, when the head of the child was at the middle strait. For organizational reasons, we could not empty the bladder in the MRI room, so we had to do so before leaving the delivery room. Most of our patients’ bladders filled during the successive examinations, and we could see that full bladders moved up above the pubic bone, whereas when they were empty, they were literally stuck between the infant’s head and the pubic bone. This is important because most teams empty the bladder just before expulsive efforts. However, this work suggests that emptying the bladder at least half an hour before beginning maternal expulsive efforts might be more protective and prevent the bladder from being dragged by the fetal head below the pubic plane.

### Comparison With Prior Work

Fetal head molding and brain shape changes during the second stage of labor were previously reported [29], but the criterion of fetal head compliance as a confounding factor in the interpretation of the presence or absence of cephalopelvic disproportion has not been sufficiently made explicit.

Regarding cephalopelvic disproportion, 2 biomechanical situations can occur during labor: the fetal head is perfectly adapted and fits the maternal birth canal without the need of molding, which occurs in eutocia; or the fetal head is of a size or geometry unsuited to pass through the birth canal and the fetal skull needs to mold to fit the maternal birth canal, which is a case of cephalopelvic disproportion. In the second case, depending on the compliance degree of the fetal head, we then observe 2 possible scenarios.

First, in deliveries with low fetal skull compliance, cephalopelvic disproportion will not allow the fetal skull to mold sufficiently to pass through the birth canal. Given that the skull bones will not overlap accordingly, uterine cervix dilatation will stop, and the labor will be defined as obstructed. The fetal head will remain high in the pelvis, and fetal skull molding will be very slow. If not resolved by a cesarean section in time, the prolonged ischemia of the fetal tissues can lead to craniotabes, caput succedaneum, and cephalohematoma; the compression of the maternal tissues can lead to necrosis or fistulas, and a postpartum hemorrhage due to uterine atony can occur. Currently, these situations can fortunately be resolved by most obstetrical teams by performing a cesarean section within 3 hours of consecutive cervix dilation stagnation, which is generally well tolerated by the fetus, except in cases of relentlessness and cesarean section performed too late or if an attempt at instrumental extraction fails. Nevertheless, traumatic delivery for the mother is frequent in these situations (red-code cesarean section, perineal tears, difficult extraction, and postpartum hemorrhage).

The deliveries presenting a cephalopelvic disproportion with high compliance are among the most puzzling because the fetal skull will adapt itself in a sugarloaf shape, thus reducing its lateral caliber in favor of an elongation in height in the occipito-bregmatic direction. The brain shape will follow this deformation. The consequences observed here are the migration of the cerebrospinal fluid from the ventricular and pericerebral spaces to the posterior fossa as well as a global folding of the cerebral mass. Thus, there are shear forces and pressure forces present, which are propelled by uterine contractions. These births can appear to be “normal deliveries” according to the usual obstetrical criteria because they will occur vaginally, most often without any identified mechanical difficulty, and they can even be rapid, with an apparent asymptomatic child. Therefore, these deliveries could appear normal for the birthing parent but be genuinely traumatic for the child and might not be identified by the current standard neonatal evaluation criteria.

The presence of these forces [28], which are exerted directly on the brain and whose effect is directly visible in imaging in our study through the morphological deformation of the skull and the brain that they induce [29], are crucial to recognize and investigate.

The cerebral handicap linked to birth is based on 2 possible events: a brain hemorrhage and a brain ischemia. Ischemia is easily detected by noting increased lactate levels and by the presence of metabolic acidosis according to pH and blood gases. Cerebral and retinal hemorrhage, on the other hand, is often minimally symptomatic in the newborn and does not alter pH or lactate. Some of these hemorrhages have radiological similarities to shaken baby syndrome, which is known to often progress to disability when occurring in a developing brain.

The cerebral hemorrhages described in the literature can reportedly occur in up to 43% of asymptomatic newborns [26,27,40] and retinal hemorrhages in up to 50% of all asymptomatic newborns [41-44]. Most of the affected newborns were born vaginally or by late cesarean section. Very few were born by scheduled cesarean section; however, the possible presence of uterine contractions even in the cesarean sections described as scheduled does not mean that they did not occur during labor. Prospective studies with a thorough analysis of these elements are needed to increase the knowledge on this subject. It is important to remember that these newborns are typically asymptomatic with the usual evaluation criteria, that is, the children do not show loss of consciousness, motricity is preserved, and there are no visible seizures, so the occurrence of these abnormalities is never routinely investigated and is, therefore, mostly ignored. The detection, study, and prevention of these hemorrhages could soon be the object of investment by national and international public health organizations.

The detection of neonatal concussion due to high-compliance cephalopelvic disproportion [28,38,39] could be improved by the identification of indirect signs of such dystocia by the obstetrical team [45], such as abnormalities of the fetal heart rate with a vagal pattern during labor [46], the presence of significant dislocation or overlap of the skull bones on examination during labor, the presence of significant sugarloaf molding of the fetal head after delivery, and the presence of a significant caput succedaneum or a cephalhematoma, sometimes with softening of the skull vault bones on touch.

It would also be necessary to reinforce the evaluation of the neurological state of the child at birth, whose behavior alone and the conservation of motricity will not alert on the presence of a brain concussion, such as after a “boxing match without knockout.” This could be achieved by the systematic application of the Sarnat score [47,48] by midwives at birth; the integration of retinoscopy without pupillary dilation in the neonatal examination by pediatricians [49]; the umbilical cord sampling by obstetricians for brain concussion markers in the cord blood, such as the S100B protein [50]; and the performance of brain imaging in the event of an abnormal value of these markers.

Our MRI observations during delivery were all performed on patients with a normal or rather large pelvis according to the usual obstetrical management criteria, which allowed us to better understand, by having been able to observe these different cases of high or low fetal head compliance dystocia, why radiological pelvic examinations alone typically fail to detect these situations effectively. It is necessary to compare the geometry and size of the child’s skull with the geometry and the dimensions of the maternal pelvis, and not to forget that the compliance of the fetal head remains for the moment an unknown criterion; therefore, the focus of interpretation should not be on the simple modality of the delivery route but on the existence or not of a traumatic delivery situation for the mother or child or both and the development of the resources required to detect these traumas.

### Limitations

Providing imaging evidence of what happens during the physiological process of birth is paramount. However, the small sample size of the populations studied with MRI during labor and delivery causes the requirement for many more prospective imaging studies (with all available modalities of medical imaging) to further explore the complexity of the biomechanical events occurring during birth.

### Conclusions

Our observations with MRI during childbirth suggest that, depending on fetal head moldability, cephalopelvic disproportion can result in either obstructed labor or apparent normal vaginal delivery with major fetal head molding and brain compression. MRI might be the best imaging technology by which to explore all the combined aspects of cephalopelvic disproportion already described by Mengert [1] and to achieve a better understanding of the underlying mechanisms of fetal head molding and moldability.

Prospective randomized cohort studies should establish a policy of routine bladder emptying earlier than at the time of expulsive efforts. Placental location could also be useful information for anticipating the presentation of the child at delivery.

The use of MRI in pregnancy and delivery creates new avenues through which to understand the physiology of the biomechanical process of childbirth and fetal adaptation during the passage to extrauterine life. The ability to share experiences related to the safety protocols that are currently being undertaken or planned using real-time MRI of the human birthing process could accelerate research and knowledge acquisition on this topic. Thus, a new era of “prenatological imaging” is emerging in a world of predictive and preventive medicine.

## Abbreviations

MR: magnetic resonance
MRI: magnetic resonance imaging
